# US Public Concerns About the COVID-19 Pandemic From Results of a Survey Given via Social Media

**DOI:** 10.1001/jamainternmed.2020.1369

**Published:** 2020-04-07

**Authors:** Lorene M. Nelson, Julia F. Simard, Abiodun Oluyomi, Vanessa Nava, Lisa G. Rosas, Melissa Bondy, Eleni Linos

**Affiliations:** 1 Department of Epidemiology and Population Health, School of Medicine, Stanford University, Stanford, California; 2 Section of Epidemiology and Population Science, Department of Medicine, Baylor College of Medicine, Houston, Texas; 3 Department of Dermatology, School of Medicine, Stanford University, Stanford, California

## Abstract

This survey study assessed public concerns about symptoms of coronavirus disease 2019 and individual actions in response to the pandemic.

In this survey study, we aimed to rapidly assess public concerns about the coronavirus disease 2019 (COVID-19) crisis^1,2^ in the United States before shelter-in-place orders were widely implemented. Specifically, we assessed concerns about COVID-19, symptoms, and individual actions in response to the pandemic.

## Methods

On March 14, 2020, we posted an online cross-sectional survey^3^ on 3 social media platforms (Twitter, Facebook, and Nextdoor) to collect data on symptoms, concerns, and individual actions taken. Twitter and Facebook posts were shareable to facilitate snowball sampling. The institutional review board at Stanford University approved this study, and informed consent was obtained from participants. Participants were informed of the purpose, risks, and benefits; minimal personal information (eg, zip code and Qualtrics location) was collected. The survey included 21 questions (multiple choice, single choice, numeric, and open ended). A paper version of the survey was pilot tested with 41 participants, who were not included in the final results. In addition to demographic data (Table), we asked about recent cold and flulike illness. Other questions addressed participants’ concerns and lifestyle modifications. All questions were optional; thus, the number of respondents to each question varied. As of March 16, 2020, 844 of 3233 (26.1%) US counties had at least 1 respondent (range, 1-868 respondents). More details about the Methods are provided in the Supplement.

**Table.** Characteristics and Responses of Participants Who Completed the Online Survey Between March 14 and 16, 2020^a^

Characteristic	No. (%)
Age, y (range) (n = 8869)	44 (18-85)
Gender, female (n = 8959)	5838 (65.2)
Race/ethnicity (n = 8828)	
White	7304 (82.7)
African American or black	100 (1.1)
Latino, Hispanic, or Spanish	372 (4.2)
Asian	778 (8.8)
Native Hawaiian or Pacific Islander	13 (0.2)
Other	261 (3.0)
What is the highest degree or level of school you have completed? (n = 7412)	
>High school	28 (0.4)
High school or General Educational Development	205 (2.8)
Some college	1243 (16.8)
Bachelor degree	2575 (34.7)
Graduate degree	3361 (45.3)
In the past 4 weeks, have you been ill with a cold or flulike illness? (n = 8982)	
No	6108 (68.0)
Yes	2874 (32.0)
To what extent are you self-isolating? (n = 8983)	
All of the time. I am staying at home nearly all the time.	1725 (19.2)
Most of the time. I only leave my home to buy food and other essentials.	4475 (49.9)
Some of the time. I have reduced the amount of times I am in public spaces, social gatherings, or at work.	2520 (28.1)
None of the time. I am doing everything I normally do.	263 (2.9)
Have you experienced any difficulties due to the coronavirus disease 2019 crisis? Select all that apply. (n = 6689)	
Reduced wages or work hours	981 (14.7)
I have lost my job	102 (1.5)
Childcare	979 (14.6)
Getting food	2154 (32.2)
Getting hand sanitizer or cleaning supplies	5403 (80.8)
Getting routine/essential medications	625 (9.3)
Transportation	302 (4.5)
Accessing health care	387 (5.8)
Other	627 (9.4)
If you were scheduled for a routine, nonurgent clinic appointment and your primary physician was not able to see you because they were sick or in quarantine, which of the following would you prefer? Please select 1. (n = 8944)	
Wait until my physician is available and reschedule an in-person visit with my own physician at a future date	3103 (34.7)
Reschedule an in-person visit with a different physician on the same day or within a few days of my original appointment	1034 (11.6)
Talk to my physician by phone for advice during the scheduled visit time	1254 (14.0)
Send in a photo and message for advice through a secure online portal and receive a call back by phone from my physician or care team	1225 (13.7)
Set up a video visit with my physician during the scheduled visit time	2328 (26.0)

^a^ Because all questions were optional and some questions were only asked based on the response to a prior question, the total number of respondents per survey question varied.

## Results

In 48 hours, from March 14 to 16, 2020, 9009 surveys were completed. The median age of participants was 44 (range, 18-85) years. Of participants who specified, 5838 of 8959 (65.2%) were women, 7304 of 8828 (82.7%) were white, and 2575 (34.7%) and 3361 (45.3%) of 7412 reported obtaining bachelor degrees and graduate degrees, respectively. Of 8982 respondents, 2874 (32.0%) reported having a cold or flulike illness in the past 4 weeks. Of 8950 respondents, 6043 (67.3%) were very or extremely concerned about COVID-19, although concern varied by age (Figure, A). Of these 8950 respondents, 8562 (95.7%) reported making changes to their lifestyle in response to COVID-19. The most common lifestyle changes were more hand washing (8336 [93.1%]), avoiding social gatherings (7963 [89.0%]), and stockpiling food and supplies (6686 [74.7%]) (Figure, B). In addition, 1725 of 8983 (19.2%) respondents were self-isolating all of the time, while 4475 (49.8%) were self-isolating most of the time and leaving the house only to buy food and essentials.

**Figure.**
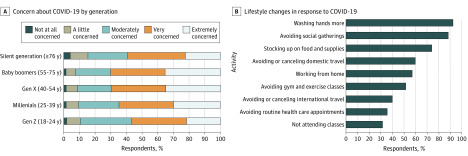
Concern About Coronavirus Disease 2019 (COVID-19) by Age Group and Lifestyle Changes Made in Response A, Responses to the question “How concerned do you feel about the novel coronavirus, COVID-19?” among each generation. Of 8891 respondents, 143 were from the silent generation, 2040 were baby boomers, 3075 were gen X, 3084 were millennials, and 549 were gen Z. B, Following the question, “Have you made any changes to your lifestyle or daily activities because of COVID-10?” respondents completed the survey question, “Which of the following are you doing? (Select all that apply).”

Top concerns participants reported were getting sick because of COVID-19 (6284 of 8966 [70.1%]) and not being able to get medical care (4149 of 8966 [46.3%]). The most common difficulties reported were obtaining hand sanitizer, food, and childcare. Among 6689 respondents, 981 (14.7%) reported reduced wages or work hours, and 102 (1.5%) reported having lost their jobs because of the crisis. If their physician was not available in person, 4807 of 8944 (53.7%) respondents indicated that they would choose to be seen via a remote televisit.

## Discussion

In this convenience sample survey distributed via 3 social media platforms in mid-March 2020, participants reported serious concerns about COVID-19 and that they were preparing by washing hands, remaining homebound, and practicing social distancing. There was variation in the levels of concern about COVID-19 in different age groups, and the most common difficulties were getting hand sanitizer or food, arranging childcare, and lost wages or reduced working hours.

We used this methodology to rapidly characterize public sentiment regarding measures taken and threats faced as the pandemic evolves while recognizing that this goal was not conducive to probability sampling. With this convenience sampling that is not representative of the public at large and lack of information on participation rates, these findings are limited and are not generalizable. Men and older individuals (who are generally known to be underrepresented on social media) were underrepresented among the respondents. Although we did not collect information on the socioeconomic status of participants, it was likely to have been high because few reported concerns about their employment. We plan additional work that will achieve greater geographic representation with focus on longitudinal trends on the health, financial, and social concerns in the United States regarding the COVID-19 pandemic.

## Notes

Supplement. **eMethods.** **eFigure. **Number of survey responses aggregated into US county boundaries. **eReferences.**

## References

[1] Thompson LA, Rasmussen SA What does the coronavirus disease 2019 (COVID-19) mean for families? JAMA Pediatr. Published online March 13, 2020. doi:10.1001/jamapediatrics.2020.0828 32167533

[2] Parodi SM, Liu VX From containment to mitigation of COVID-19 in the US. JAMA. Published online March 13, 2020. doi:10.1001/jama.2020.3882 32167525

[3] Stanford coronavirus study. https://pcrt.stanford.edu/covid. Accessed March 18, 2020.

