# Supplementary material for: US Public Concerns About the COVID-19 Pandemic From Results of a Survey Given via Social Media
Source: JAMA Intern Med. 2020 Apr 7;180(7):1020–2. doi: 10.1001/jamainternmed.2020.1369 (PMC7139509; doi:10.1001/jamainternmed.2020.1369)
Supplement: Supplement. — eMethods. eFigure. Number of survey responses aggregated into US county boundaries. eReferences. [file jamainternmed-180-1020-s001.pdf]

Supplementary Online Content

Nelson LM, Simard JF, Oluyomi A, et al. US public concerns about the COVID-19 pandemic from results of a survey given via social media. *JAMA Intern Med*. Published online April 7, 2020. doi:10.1001/jamainternmed.2020.1369

eMethods.

eFigure. Number of survey responses aggregated into US county boundaries

eReferences.

This supplementary material has been provided by the authors to give readers additional information about their work.

© 2020 Nelson LM et al. *JAMA Internal Medicine*.

Supplement

US Public Concerns About the COVID-19 Pandemic: Findings From a Survey via Social Media

eMethods

Between March 14-16 2020 (a 48-hour period), we posted an online web-based cross-sectional survey on three social media platforms (Twitter, Facebook, and NextDoor) to collect information on the current health status, concerns about Covid-19, and personal actions taken in response to the pandemic threat. Participants were informed of the purposes of the survey (including potential risks and benefits) and were asked to provide limited personal information regarding geographic location (e.g., zip code) so that we could determine how survey responses varied by geographic location.

In addition to the analyses presented in manuscript Table and Figure, we assessed the geographic distribution of our sample. We used choropleth maps to display survey data at the county level using ArcGIS Pro 2.5 (Esri, Redlands, CA)., and used the “Natural Breaks Classification” method in ArcGIS (Jenks Optimization method)[1] to show the distribution of survey responses across the US; this method minimizes the sum of the variance within each of the classes being created and maximizes the variance between classes.[2]

Supplemental Figure 1 shows the number of respondents according to geographic location. As we did not use a probability-based sampling method, our results are not generally representative of the public at large. Relative to their

© 2020 Nelson LM et al. *JAMA Internal Medicine*.

proportions in the US population, our survey underrepresents men and older individuals, groups that are generally known to be less frequent users of social media platforms. Moreover, the respondents to our survey do not represent the full diversity of the U.S. population with respect to race and ethnicity (i.e., Hispanic/Latino ethnic origin).

© 2020 Nelson LM et al. *JAMA Internal Medicine*.

eFigure. Number of survey responses aggregated into US county boundaries.

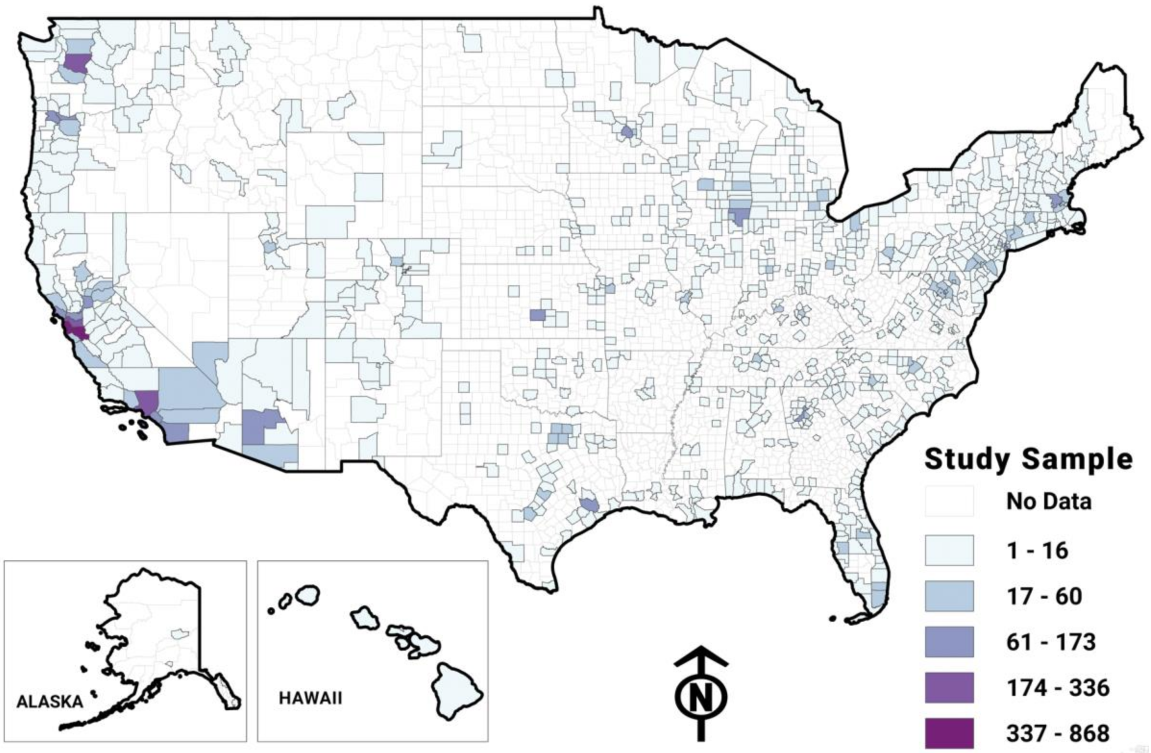

eReferences

1. McMaster, R., In Memoriam: George F. Jenks (1916-1996). Cartography and Geographic Information Science;1997:24(1): 56-59.
2. Murray, A.T. and T.K. Shyy. Integrating attribute and space characteristics in choropleth display and spatial data mining. International Journal of Geographical Information Science;2000: 14(7): 649-666.

© 2020 Nelson LM et al. *JAMA Internal Medicine*.
